# PET/MR and SPECT/MR multimodal imaging constructs: Direct radiolabelling of silica shell iron oxide nanorods for use in liver imaging and potential for hyperthermia therapy

**DOI:** 10.1186/2197-7364-2-S1-A87

**Published:** 2015-05-18

**Authors:** Haylay Bignell

**Affiliations:** University of Hull, UK

Superparamagnetic iron oxide nanoparticles (SPIONs) are used as T2 magnetic resonance (MR) contrast agents. Nanorods (NRs) offer an interesting alternative to the more widely used nanospheres as they have shown to offer enhanced T2 relaxivities. The combination of MRI with nuclear imaging modalities such as positron emission tomography (PET) or single photon emission computed tomography (SPECT) increases the data available from a single diagnostic scan (e.g. quantification, multiple image overlay). Radiolabelling of SPIONs allows high sensitivity nanoparticle biodistribution data which can both aid in future construct design and be used directly for precise liver lesion imaging. In this work we report the synthesis and characterisation of silica shell iron oxide NRs functionalised with varying ratios of polyethylene glycol (PEG) and the tetraazamacrocyclic chelator, DO3A. Direct and facile radiolabelling of the constructs with the radioisotope gallium-68 (t1/2 = 68 min) proceeded with quantitative radiochemical yields in 15 min and no evidence of radioisotope dissociation was observed after 3 h in both serum and in competition with apo-transferrin. Interestingly, it was observed that neither the radiolabelling process nor stability in vitro or in vivo was compromised by the absence of the bifunctional chelating moiety. Consequently silica shell NRs with 100 % PEG coating were evaluated for potential use as SPECT/MR imaging agents; direct radiolabelling with technetium-99m (t1/2 = 6.02 h) proceeded with analogous radiochemical yields and stabilities. In vivo imaging studies showed rapid liver uptake with high T2 contrast, demonstrating the application of silica shell iron oxide NRs as bimodal PET/MR and SPECT/MR liver imaging agents. Preliminary magnetic hyperthermia evaluation indicates the potential future use of the constructs developed as multimodal theranostic agents.

